# Recent Advances in Mass Spectrometry-Based Protein Interactome Studies

**DOI:** 10.1016/j.mcpro.2024.100887

**Published:** 2024-11-27

**Authors:** Shaowen Wu, Sheng Zhang, Chun-Ming Liu, Alisdair R. Fernie, Shijuan Yan

**Affiliations:** 1 State Key Laboratory of Swine and Poultry Breeding Industry, Guangdong Key Laboratory of Crop Germplasm Resources Preservation and Utilization, Agro-biological Gene Research Center, Guangdong Academy of Agricultural Sciences, Guangzhou, China; 2 Proteomics and Metabolomics Facility, Institute of Biotechnology, Cornell University, Ithaca, New York, USA; 3 Key Laboratory of Plant Molecular Physiology Institute of Botany, Chinese Academy of Sciences, Beijing, China; 4 Root Biology and Symbiosis, Max Planck Institute of Molecular Plant Physiology, Potsdam-Golm, Germany

**Keywords:** mass spectrometry, protein interactome, affinity purification, proximity labeling, cross-linking, co-fractionation, computational tools

## Abstract

The foundation of all biological processes is the network of diverse and dynamic protein interactions with other molecules in cells known as the interactome. Understanding the interactome is crucial for elucidating molecular mechanisms but has been a longstanding challenge. Recent developments in mass spectrometry (MS)-based techniques, including affinity purification, proximity labeling, cross-linking, and co-fractionation mass spectrometry (MS), have significantly enhanced our abilities to study the interactome. They do so by identifying and quantifying protein interactions yielding profound insights into protein organizations and functions. This review summarizes recent advances in MS-based interactomics, focusing on the development of techniques that capture protein-protein, protein-metabolite, and protein-nucleic acid interactions. Additionally, we discuss how integrated MS-based approaches have been applied to diverse biological samples, focusing on significant discoveries that have leveraged our understanding of cellular functions. Finally, we highlight state-of-the-art bioinformatic approaches for predictions of interactome and complex modeling, as well as strategies for combining experimental interactome data with computation methods, thereby enhancing the ability of MS-based techniques to identify protein interactomes. Indeed, advances in MS technologies and their integrations with computational biology provide new directions and avenues for interactome research, leveraging new insights into mechanisms that govern the molecular architecture of living cells and, thereby, our comprehension of biological processes.

## Highlights

• Recent advances in MS-based techniques for comprehensive protein interactome mapping.
• Integration of experimental MS methods with cutting-edge computational tools.
• Emerging strategies for high-resolution interactome analysis in various biological contexts.

## Graphical Abstract

**Figure:**
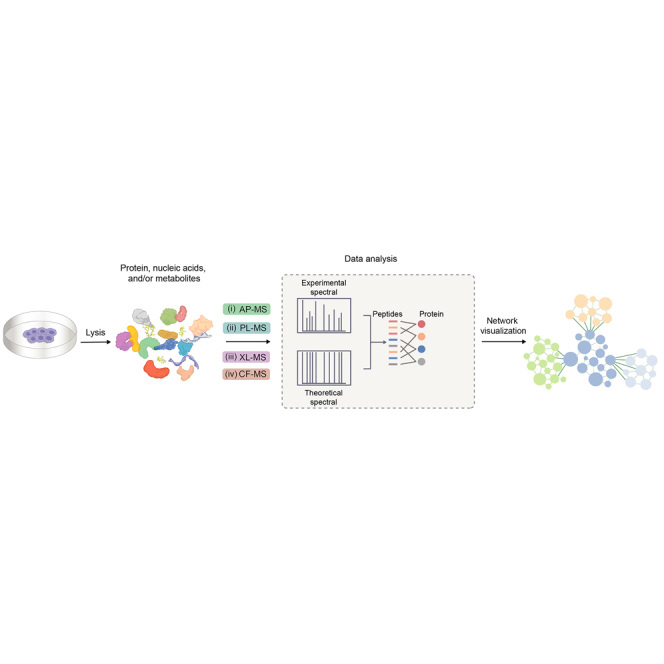

## In Brief

This review highlights recent advancements in mass spectrometry-based techniques for mapping protein interactomes, including affinity purification, proximity labeling, cross-linking, and co-fractionation approaches. It discusses the integration of these methods with cutting-edge computational tools, emphasizing their synergistic potential in elucidating complex cellular networks. The review explores how these technologies are revolutionizing our understanding of protein interactions, providing unprecedented insights into cellular processes and opening new avenues for biological research, drug discovery, and disease diagnostics.

Proteins primarily regulate cellular processes and physiological activities through complex interactions with other proteins, metabolites, and nucleic acids that are important for normal cellular functions (1, 2, 3). These interactions are vast. For example, broad-scale screens using human cells have suggested the presence of up to 130,000 binary protein-protein interactions at any given time, with the added number of protein-metabolite and protein-nucleic acids being likely to far exceed this number (4, 5). This framework delineates the recognized roles for protein-protein interactions (PPIs), including the formation of protein complexes that modulate cellular signaling, gene regulation, and structural organization (6, 7). Meanwhile, protein-metabolite interactions (PMIs) regulate enzyme kinetics, configure metabolic pathways, and maintain cellular energy homeostasis in processes of major consequence for metabolic regulation (8, 9). While protein-nucleic acid interactions (PNIs) orchestrate a wide variety of genetic processes, including transcription, translation, RNA splicing, and chromatin remodeling, all important for the dynamic control of gene expression and genomic stability (3, 10). The protein interactome comprehensively maps the totality of protein-based molecular connections and is hence vital for a full understanding of biological functions (1, 2).

Investigation of protein interactions has traditionally relied on techniques such as yeast two-hybrid and one-hybrid assays. The yeast two-hybrid (Y2H) system and its derivatives, such as mammalian two-hybrid systems, offer distinct advantages including detection of binary protein-protein interactions, high-throughput capability for large-scale screening, and ability to detect weak interactions (11, 12). Similarly, the yeast one-hybrid (Y1H) system applies analogous principles to study protein-DNA interactions, particularly useful for identifying transcription factors that bind to specific DNA sequences (13). However, these yeast-based approaches are limited to detecting binary interactions and have several drawbacks including both high false-positive rate and false-negative rate, the requirement of targeted proteins being stable in the yeast cell, independent of post-translational modifications, and failure to detect short-lived interactions with low-abundance proteins, or transient in nature (12).

By contrast, over the last 2 decades, mass spectrometry (MS)-based proteomics has dramatically expanded both the scale and depth of interactome analysis (1, 2, 3, 6, 7). This technological evolution has brought various methodologies such as affinity purification-MS (AP-MS), proximity labeling-MS (PL-MS), cross-linking-MS (XL-MS), and co-fractionation-MS (CF-MS) (6, 7, 14, 15), into play. AP-MS provides detailed interactome maps by isolating protein complexes using specific affinity tags, with the bait protein could be expressed at near-physiological conditions (16). PL-MS methods such as BioID and TurboID allow for the study of protein interactions within their native cellular contexts and capture transient interactions through covalent biotinylation tagging (15). XL-MS provides structural insights by stabilizing interactions via chemical cross-linkers for distance restraints critical for understanding both spatial relationships and interaction domains (17). CF-MS allows for the resolution of protein complexes fractionated according to biophysical properties, followed by MS analysis (18).

By integrating innovative sample preparation strategies, enrichment techniques, high-resolution MS instrumentation, and sophisticated computational frameworks, these methods have enabled system-wide charting of PPIs to an unprecedented depth and, although PMIs and PNIs are more challenging to capture due to the transient nature of metabolites and the complexity of nucleic acid interactions, progress has also been made in these areas, collectively providing considerable insights into the intricate networks that govern cellular life (3, 8, 18, 19, 20, 21, 22, 23). As these MS-based approaches revolutionize interactome studies, it remains essential to complement them with orthogonal validation methods. Techniques such as Y2H, Y1H, bimolecular fluorescence complementation (BiFC), Förster resonance energy transfer (FRET), bioluminescence resonance energy transfer (BRET), split-luciferase assays, surface plasmon resonance (SPR), and microscale thermophoresis (MST) play a critical role in confirming and further characterizing interactions identified through MS-based approaches (24, 25).

Furthermore, recent advances in MS-based proteomics techniques, which integrate computational biology with MS have become pivotal in interactome analyses, substantially enhancing our understanding of complex molecular interactions within their cellular contexts. Computational tools have become indispensable in dealing with the large torrents of data produced by MS both to elucidate otherwise obscure patterns and relationships and to aid in the reconstruction of interaction networks (26). Advanced computational algorithms are increasingly used to facilitate MS data analysis, thereby enhancing the accuracy of protein identification and validation of interactions (27, 28, 29, 30). Besides, computational modeling allows a structural perspective of protein complexes identified by MS, facilitating predictions about their three-dimensional structures and functional implications (31, 32).

The recent advances in MS-based techniques have revolutionized our ability to elucidate the intricate details of protein interactions, enabling comprehensive and in-depth analysis of protein networks across all domains of life. This review attempts to present an overview of recent developments, focusing on developing and refining protein interactomics methodologies related to MS technology for various fields in biological research. The growing role of computational tools has been particularly emphasized in enhancing MS-based analysis, predicting protein interactions, and modeling complexes. Additionally, we examine the integration of MS-based approaches with advanced computational methods, showcasing how these synergistic strategies are reshaping our understanding of cellular processes. By discussing current challenges and future prospects, this review aims to stimulate further innovation in interactome research, ultimately deepening our comprehension of fundamental biological mechanisms.

## Mass Spectrometry-Based Techniques for Investigation of Large-Scale Protein Interaction Networks

MS-based techniques, including (i) AP-MS, (ii) PL-MS, (iii) XL-MS, and (iv) CF-MS, have dramatically expanded our understanding of protein interactions in biological systems. The technical details of these methodologies have been thoroughly described in several previous research papers (33, 34, 35, 36). Building on previous foundational work, recent developments in these techniques have significantly enhanced their effectiveness and widened their scope of applications, proving essential in basic biological studies. Herein, we highlight the recent development of these methodologies and their diverse applications over the last 5 years, emphasizing their importance in both comprehensive interactome mapping and complex protein network analysis. In addition, MS-based methods such as thermal protein profiling (TPP) and limited proteolysis coupled to MS (LiP-MS) have also been employed to characterize protein interactomes (37, 38). For example, we previously utilized TPP to identify the interaction between autoxidated linolenic acid (AL) and proteins in *Aspergillus flavus*, revealing that AL interacts with a transporter protein ImqJ located in the imizoquin (IMQ) biosynthetic pathway, as well as α-tubulin, RNA helicase, and short-chain oxidoreductase (39). These methodologies have been reviewed extensively in other publications (37, 38), and thus, we refer readers to those reviews for more detailed information.

### AP-MS

**Figure 1:**
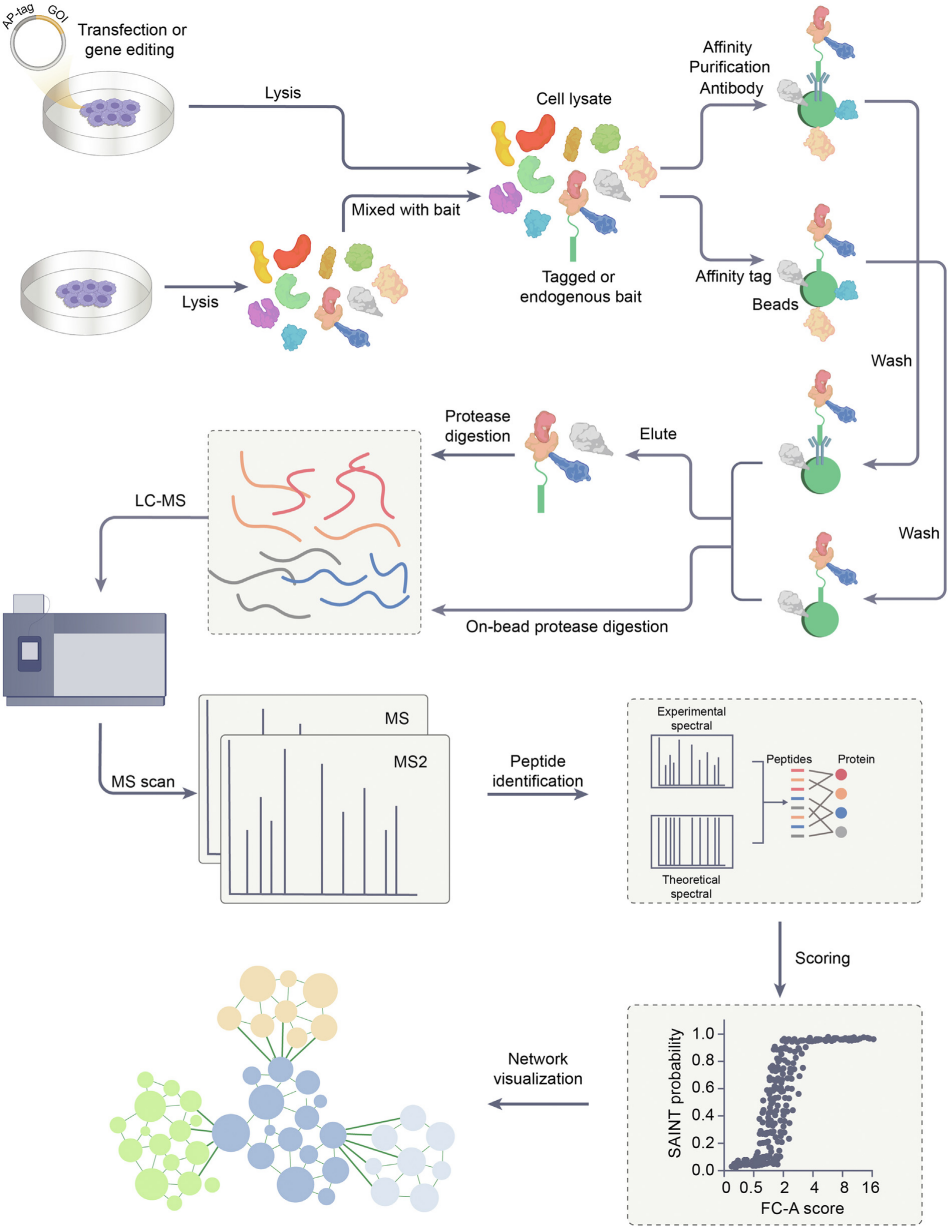
**The workflow of AP-MS.** The process can begin with transfection, gene editing of a gene of interest (GOI), or incubation to introduce the bait molecule, followed by cell lysis to obtain the cell lysate, or directly with cell lysis when working with labeled baits, then incubating the bait with the cell lysate. The bait can be a protein, nucleic acid, or metabolite, and is often tagged or linked to an appropriate label for its subsequent purification. For proteins, an affinity tag or antibody is most commonly used. Nucleic acids are typically labeled with biotin, or sequences can be recognized by antibodies. Metabolites, on the other hand, are usually coupled with a reactive group. During the sample preparation process, the bait molecule interacts with the proteins in the cell or is incubated with a cell lysate containing a complex mixture of proteins. After allowing sufficient time for these interactions to occur, the bait and its bound interaction partners are captured using an affinity matrix (beads) that specifically binds to the bait's label or an antibody against the bait. Stringent wash steps are performed to reduce non-specific binding, however, complete removal of background proteins is not possible. At this point, the workflow presents two common paths for protein digestion: the bait, its specific interacting partners, and some residual non-specific proteins are eluted from the affinity matrix and then subjected to protease digestion, or alternatively, on-bead protease digestion may be performed, which can result in higher background. Following digestion, the resulting peptides are analyzed by LC-MS/MS. The analysis begins with an MS scan to collect raw mass spectrometry data and peptide identification is then performed by comparing experimental and theoretical spectra, after which the identified proteins may be scored using computational algorithms (49, 50). Subsequent data analysis is crucial to distinguish true interactors from background contaminants among the hundreds to thousands of proteins typically identified in modern AP-MS experiments. The resulting protein interactions are mapped into a network for visual representation and interpretation.

Affinity purification-mass spectrometry (AP-MS) is a robust technique for elucidating protein interactions by coupling affinity purification with MS analysis. In a typical AP-MS procedure, a(n often tagged) molecule of interest (bait) is selectively enriched along with its associated interaction partners (prey) from a complex biological sample using an affinity matrix, such as an antibody raised either against a specific bait or a specific tag (Fig. 1) (16, 40, 41). The bait-prey/tag complexes are subsequently washed with high stringency to remove non-specifically bound proteins and then eluted from the affinity matrix. The purified proteins are digested into peptides, either on-bead or after elution, and then labeled with tandem mass tags (TMT) or subjected to label-free quantitation via liquid chromatography-mass spectrometry (LC-MS/MS) (42). In this way, it is possible to identify prey proteins that are associated with a particular bait. The resulting data undergoes computational analysis to distinguish true interactors from background contaminants. However, the success and reliability of this technique are largely determined by the selection of affinity purification methods, the experimental design (e.g., appropriate controls), and the data analysis approach.

A critical decision in designing an AP-MS experiment is whether to use antibodies against endogenous proteins or to employ tagged proteins for affinity purification. This choice is crucial as each approach has its own set of advantages and limitations. Antibodies against endogenous proteins offer the advantage of studying proteins in their native state but can be challenging to generate with high specificity (43). On the other hand, tagging the bait protein allows for more standardized purification but introduces its own challenges. When opting for tagged proteins, researchers must further choose between overexpression of tagged proteins or endogenous tagging using genome editing techniques like CRISPR-Cas9. Overexpression of tagged bait protein can lead to non-physiological protein levels, potentially causing artifacts in the detected interactions (24). In contrast, CRISPR-Cas9-mediated endogenous tagging maintains native expression levels but can be technically challenging and time-consuming (44). Furthermore, the addition of a tag may interfere with protein folding, localization, or interaction interfaces, particularly for smaller proteins or those with critical C- or N-terminal interaction domains (45). These considerations highlight the complexity of designing effective AP-MS experiments and the need for careful validation of results. The complexity of AP-MS experiments necessitates careful consideration of experimental design and data analysis to ensure reliable results. Hence, proper controls, such as negative controls with non-expressing cells or competitive controls with excess free bait, are essential to distinguish true interactors from background contaminants.

To mitigate some issues that were prevalent in studies of the past decade, including a high false-positive rate due to nonspecific binding, a high false-negative rate for the potentially low abundance level of binding partners, complex sample preparation, and difficulty in detecting transient interactions, several innovative AP-MS methods have been developed recently. Spin-tip columns and novel affinity tags have been utilized to enhance the efficiency of AP-MS. For example, monolithic micro immobilized metal ion affinity chromatography columns (m-IMAC) prepared in pipette tips (spin-tip columns) have improved the efficiency of affinity purification and enabled sensitive label-free quantitative proteomics (46). The Fully Integrated Spin-Tip AP-MS (FISAP) system consolidates all AP-MS steps into a single spin-tip column, significantly improving protein profiling efficiency from minimal lysate volumes (42). The novel HiP4 (histidine plus four amino acids) tag system allows a tandem affinity purification (TAP) by integrated use of nickel bead purification with HiP4 tag immunoprecipitation and provides comprehensive protein-protein interaction analyses with low background and high selectivity (16).

Additional techniques have been developed, building on the innovations mentioned above, to address more complicated scenarios. For example, to tackle the issue of unresolved isoforms concurrently present in the AP sample, deep interactome profiling by mass spectrometry (DIP-MS) was developed (40). This method combines affinity purification with blue-native-PAGE separation and advanced signal processing, significantly enhancing the resolution of complex protein interactions. Furthermore, a sensitive high-throughput affinity enrichment coupled to the MS method was developed, employing a quantitative two-dimensional analysis strategy that enabled comprehensive mapping of the *Saccharomyces cerevisiae* interactome, revealing near saturation level networks with significant improvements in detecting complex interactions (47). Triplexed affinity reagents, employing metabolically stable inositol pyrophosphate analogs, were developed to systematically annotate the mammalian interactome of inositol pyrophosphate 5PP-InsP(5) (48). Additionally, data analysis methods like Significance Analysis of INTeractome (SAINT) and Contaminant Repository for Affinity Purification (CRAPome) employ statistical approaches to score and filter interactions, helping to minimize false positives and identify high-confidence protein interactions (49, 50). These developments have expanded the potential of AP-MS, allowing for deeper and more comprehensive insights into the dynamic interactomes of complex biological systems (Table 1).

**Table 1:** AP-MS approaches for protein interactomics

Variations	Features	Strengths	Limitations	Use cases	Ref
Tandem AP-MS	Uses double affinity tags/antibodies for purification	Offers higher specificity	May miss weak or transient interactions; has more complex protocol	Stable protein complexes; high confidence interactome mapping	(16)
Miniaturized AP-MS	Includes FISAP and m-IMAC spin-tip columns	Improved efficiency, works with small sample volumes	May have limitations for large-scale studies	Rapid screening of interactions, sensitive label-free quantitative proteomics	(42, 46)
Specialized AP-MS	e.g., Triplexed affinity reagents for metabolite interactomes	Enables systematic annotation of specific interactomes	Limited to specific molecule classes or applications	Studying specific molecular interactomes (e.g., inositol pyrophosphate)	(48)

AP-MS has been extensively utilized across various research fields, including signal transduction, gene regulation, assembly of protein complexes, and enzyme-enzyme associations, including metabolons (also known as substrate channels) (51, 52, 53, 54, 55). For example, this technique was recently used to successfully discover ribosome-associated proteins in chloroplasts, as well as to identify regulatory factors critical for protein synthesis and complex assembly (53). The dynamics of signalosome assembly around canonical proteins of the T-cell receptor signaling pathway were subsequently analyzed using AP-MS in primary T cells, revealing that the TCR signal-transduction network comprises at least 277 unique proteins involved in 366 high-confidence interactions while TCR signals diversify extensively at the level of the plasma membrane (56). AP-MS has proven to be a pivotal methodology in plant systems for elucidating metabolons, multi-enzyme complexes that orchestrate substrate channeling and modulate metabolic fluxes. Utilizing AP-MS, we have dissected the tricarboxylic acid (TCA) cycle metabolon, integrating quantitative AP-MS data with the findings from split-luciferase and yeast-two-hybrid assays to establish a statistical reliability score for evaluating protein-protein interactions (55). This approach unveiled 158 interactions, including those between catalytic subunits of sequentially acting enzymes and between subunits of enzymes that catalyze non-adjacent reactions. However, it is important to note that AP-MS alone cannot define a metabolon but merely an enzyme-enzyme interaction. By amalgamating AP-MS with other binary techniques such as bimolecular fluorescence complementation and Förster resonance energy transfer, the dynamics of interactions within the glycolytic metabolon have been elucidated under physiologically pertinent energy conditions. Collectively, these investigations underscore the robustness of AP-MS as a method for delineating enzyme-enzyme assemblies both for constitutive multi-enzyme complexes and dynamic enzyme-enzyme interactions, which have been summarized more comprehensively in our previous reviews (57, 58).

While AP-MS has been instrumental in elucidating protein-protein interactions, its applications extend to a broader spectrum of molecular interactions. By adopting the core principles of affinity purification, researchers have expanded AP-MS to explore protein interactions involving metabolites and nucleic acids, thus providing a more comprehensive view of the cellular interactome. Employing 2′,3′-cyclic AMP (2′,3′-cAMP) as bait, AP-MS identified the interactions of this metabolite with the stress granule (SG) proteome, revealing Rbp47b as a crucial interacting partner and greatly advancing the understanding of 2′,3′-cAMP’s role in plant stress responses (23). A high-throughput strategy combining tobramycin affinity purification and MS was developed to characterize the interacting proteins of the long non-coding RNA HULC, revealing its role in promoting aerobic glycolysis (52). Moreover, anti-dsRNA immunoprecipitation followed by MS analysis was used to characterize the dsRNA interactome in Sindbis virus-infected human cells, identifying SFPQ as a novel proviral dsRNA-binding protein (59). These studies underscore the value of AP-MS in exploring various molecular interaction networks and advancing our understanding of diverse biological processes, while also highlighting its adaptability through the development of various techniques for investigating different types of molecular interactions.

### PL-MS

**Figure 2:**
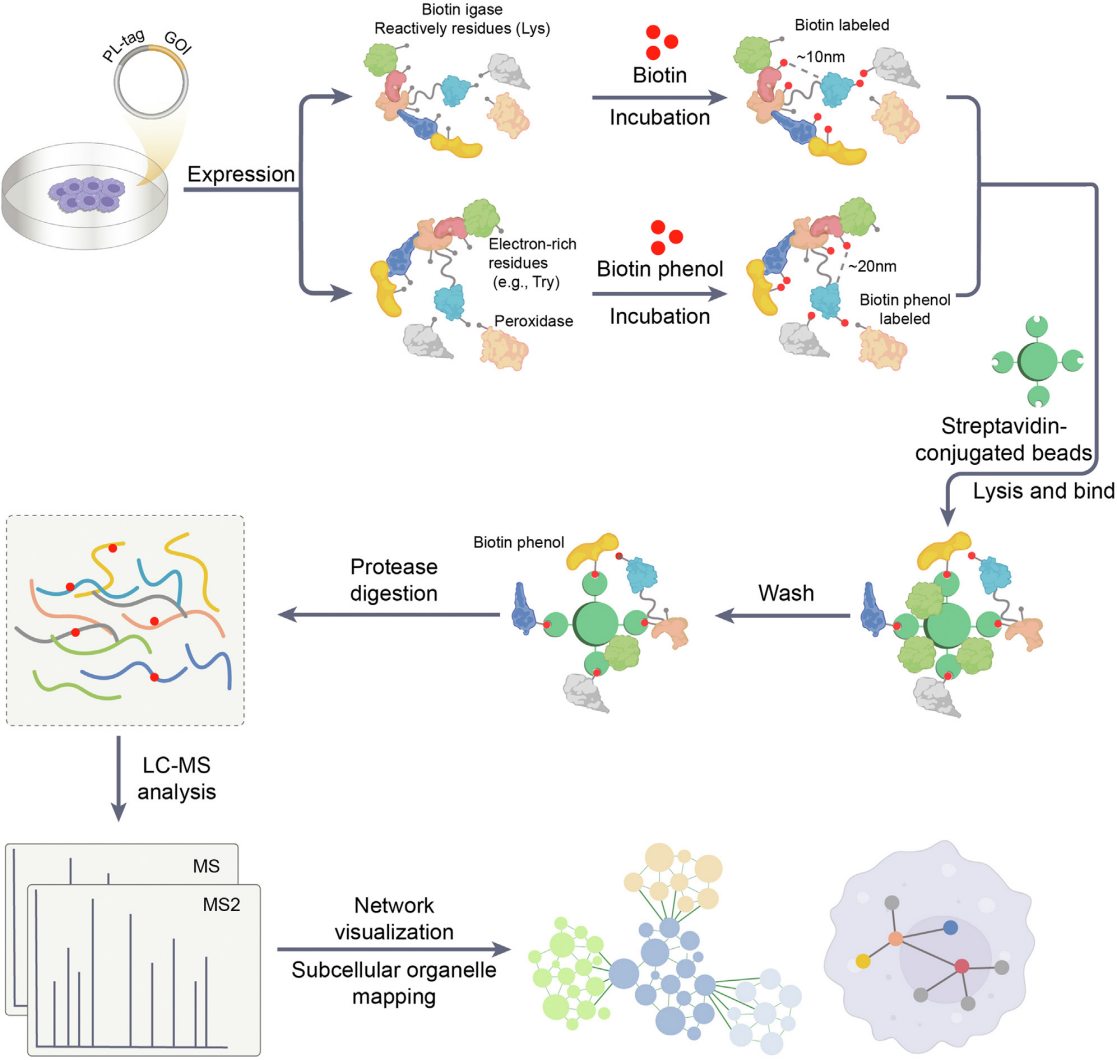
**The workflow of PL-MS.** A chosen bait molecule is coupled to a biotin ligase (BioID) or a peroxidase (APEX). The coupling strategy varies depending on the bait type: for proteins, the labeling enzyme is directly fused; for nucleic acids, the labeling enzyme is fused to a protein that binds specific nucleic acid structures or modification sites; and for metabolites, the labeling enzyme is typically fused to a receptor that binds the metabolite or its variants. *In vivo* labeling is initiated by incubating cells with biotin (for BioID) or biotin phenol (for APEX), which are taken up by cells and converted into reactive intermediates by the respective labeling enzymes. In a distance-dependent way, these reactive intermediates diffuse from the labeling enzyme to covalently modify lysine (Lys) residues (for BioID) or tyrosine (Tyr) residues (for APEX), which are located within approximately 10 nm (for BioID) or 20 nm (for APEX) from the bait molecule. After the labeling period, cells are lysed under harsh denaturing conditions to disrupt cellular structures. Biotinylated proteins, including the bait molecule and its proximal interacting partners, or proximal proteins without direct or indirect interaction, are then captured using streptavidin-conjugated beads, thus preserving bait-prey interactions. Following washing steps to remove non-specifically bound proteins, the biotinylated proteins are either eluted for subsequent tryptic digestion or subjected to on-bead digestion using trypsin-resistant streptavidin beads. The resulting peptides are then analyzed by LC-MS/MS. Subsequent data analysis involves MS and MS2 scans for peptide identification, followed by network visualization and subcellular organelle mapping to interpret the protein interaction landscape and localization of the bait and its interacting partners.

The development of proximity labeling-mass spectrometry (PL-MS) has been driven by the need for techniques that capture transient and weak interactions with a low abundance of binding partners. Such interactions are often lost during conventional AP methods or under the detection limit of nanoLC-MS/MS. Nowadays, PL-MS has emerged as a powerful technique for delineating protein interactions and characterizing cellular complex compositions within their native environments. In this approach, a protein of interest (bait) is fused to an engineered labeling enzyme with broad substrate specificity, such as a biotin ligase or peroxidase. This bait-enzyme fusion is typically overexpressed within living cells through transfection or stable cell line generation. In some cases, it may be expressed at endogenous levels using genome editing techniques. Cells or lysates are then incubated with the enzyme's substrate (biotin for biotin ligases, biotin-phenol for peroxidases), which is converted to reactive intermediates. These reactive intermediates covalently tag proximal proteins (prey) within about 10 to 20 nm range of the bait (Fig. 2) (1, 60). Following the labeling period, cells are lysed under denaturing conditions to solubilize proteins and inactivate labeling enzymes. Biotinylated proteins are subsequently captured using streptavidin-conjugated beads and subjected to stringent washes to remove non-specifically bound proteins. The enriched proteins are then eluted from the beads and enzymatically digested into peptides. These peptides are analyzed by LC-MS/MS for identification and quantification, followed by bioinformatics analysis to identify bait-proximal proteins and construct interaction networks, referred to as the proximal proteome, which includes but is not limited to the bait's interactome.

The choice of labeling enzyme is a pivotal factor in PL-MS studies, with each option offering distinct advantages. One of the pioneer PL-MS methods, BioID, utilized a mutated form of the *Escherichia coli* biotin ligase BirA (BirA∗) that catalyzes the biotin and adenosine triphosphate (ATP) to produce a reactive intermediate, biotin–adenosine monophosphate (biotin-5ʹ-AMP), which allows to biotinylate neighboring proteins *in vivo* (61). Despite its utility, the slow kinetics of BioID catalysis (high affinity and specificity for biotin-5ʹ-AMP) resulted in prolonged labeling times, prompting the development of more efficient biotin ligases. Notably, TurboID, featuring mutations that boost activity and substrate promiscuity, has enhanced the spatial and temporal resolution of proximity labeling, though it may also increase the risk of non-specific tagging (33). Further innovations have yielded even more compact and highly active labeling enzymes, such as miniTurbo, suitable for various applications, including bacterial systems (62). Recent advancements have introduced ultraID and microID, which are smaller and more potent variants of BioID, thereby increasing the flexibility of biotin ligase-based PL-MS (63).

Meanwhile, peroxidase-based PL-MS methods utilizing the catalytic prowess of engineered ascorbate peroxidases like APEX2 have been developed (64). APEX2 catalyzes the oxidation of biotin-phenol to the short-lived biotin-phenoxyl radical under the presence of hydrogen peroxide. This radical reacts with electron-rich tyrosine on neighboring proteins, leading to their biotinylation of adjacent proteins in a shorter timeframe, thus enabling the detection of highly transient interactions. Strikingly, this APEX-based PL-MS approach has recently emerged as a revolutionary approach to reveal the subcellular proteomes (65). The APEX2 proximity ligation method can be used to identify RNA, protein, and DNA interacting with or in proximity to the nuclear lamina; however, the need for hydrogen peroxide may interfere with normal cellular processes (66). Recent advancements have introduced a novel method called Photoactivation-Dependent Proximity Labeling (PDPL). This technique employs the photosensitizer protein miniSOG, which generates singlet oxygen upon exposure to blue light, allowing for high-fidelity labeling of protein networks with both spatial and temporal resolution (67). PDPL has demonstrated greater specificity and deeper proteomic coverage compared to existing methods, successfully identifying organelle-specific proteomes and novel interactors of disease-related proteins like cancer-related epigenetic regulator protein BRD4 and Parkinson's disease-related E3 ligase Parkin (67). The evolution of enzyme labeling techniques in PL-MS has led to increasingly efficient, versatile, and spatiotemporally precise methods for studying protein interactions and cellular proteomes, with each approach offering unique advantages for specific research applications (Table 2).

**Table 2:** PL-MS strategies for protein interactome mapping

Variations	Features	Strengths	Limitations	Use cases	Ref
BioID-based	Includes BioID, TurboID, miniTurbo, ultraID, microID	Works in living cells, captures weak interactions; newer variants offer faster labeling and higher efficiency	Potential for background labeling; kinetics vary between variants	Mapping interactomes in live cells; time-course studies; studies requiring minimal perturbation	(33, 61, 62, 63)
APEX-based	Includes APEX, APEX2, and variations for nucleic acid proximity labeling	Very fast labeling, high spatial resolution; can identify interactions with multiple biomolecule types	Requires H_2_O_2_ addition, potential oxidative stress	Capturing transient interactions, subcellular proteomics; comprehensive biomolecular interaction studies	(64, 65, 66)
Photo-activatable	e.g., Photoactivation-Dependent Proximity Labeling (PDPL)	High-fidelity labeling with spatial and temporal resolution	Requires light exposure, potential photo-damage	Studying dynamic protein interactions with high specificity	(67)

The efficacy of PL-MS studies hinges on careful experimental design and robust data analysis. In addition to the choice of labeling enzyme, optimizing labeling conditions is crucial, researchers must balance labeling duration and biotin concentration to maximize true interactions while minimizing false positives. It is also important that the expression levels of the bait protein-enzyme fusion should mimic physiological conditions to avoid artifacts. Stringent controls, such as expressing the labeling enzyme alone without fusion to the bait protein, are essential to establish a baseline for non-specific labeling and to distinguish genuine proximity-dependent interactions from background labeling events. By carefully considering these factors, researchers can maximize the effectiveness of PL-MS to uncover transient and weak interactions that often elude traditional methods, providing unprecedented insights into the spatial organization of protein complexes in living cells, as exemplified by its adaptability and applications across various biological systems and research domains. For example, a super-resolution proximity labeling (SR-PL) method, which utilizes a versatile "plug-and-playable" PL enzyme, TurboID-GBP (GFP-binding nanobody protein), was recently introduced (68). This technique was employed to map the interactomes of SARS-CoV-2 ORF3a and membrane protein (M), identifying 224 and 272 peptides as ORF3a and M interactors, respectively. PL-MS has been successfully applied in plant systems like *Lotus japonicus* and *Arabidopsis thaliana* (60, 69). In *Caenorhabditis elegans*, the depletion of naturally biotinylated carboxylases has been shown to dramatically reduce the background and increase the sensitivity of TurboID-based proximity labeling, facilitating a more thorough investigation of protein interactions (70). Furthermore, immunoproximity biotinylation (IPL) has emerged as a powerful approach for proximity labeling. This technique uses an antibody to guide biotin deposition onto adjacent proteins in cells and primary tissues, allowing for the capture and identification of proximal proteins by MS (71). IPL has been applied to target the axon initial segment (AIS) proteome by utilizing antibodies to restrict horseradish peroxidase (HRP) to the AIS, enabling the identification of AIS proteins in fixed cortical neurons (72). IPL-AIS has revealed the dynamic changes in AIS components during neuronal maturation and identified previously unreported AIS proteins, such as tumor-suppressor protein scribble SCRIB, which interact with ankyrin-G and are crucial for AIS structure and function (72).

The proximity-based labeling approach of PL-MS has also been valuable for investigating protein interactions with other biomolecules. By creatively adapting PL-MS techniques, researchers have opened new avenues for studying protein-RNA interactions through methods like RNA-BioID, further expanding our understanding of complex cellular processes (73, 74). The Genomic Locus Proteomics (GLoPro) method combines CRISPR-based genome targeting, proximity labeling, and quantitative proteomics to discover proteins associated with specific genomic loci in native cellular contexts (75). RNA-centric proximity labeling approaches utilizing catalytically inactive Cas13 variants (dCas13) have enabled the identification of dynamic RNA-protein interactions in living cells, revealing novel RNA binding proteins and regulatory complexes (76). Hybridization-proximity labeling techniques such as HyPro-MS and HyPro-seq have enabled the exploration of proteins and transcripts associated with specific RNAs, illuminating the functional architecture of nuclear RNA compartments (77). Furthermore, PL-MS has proven instrumental in investigating small molecule interactomes, as demonstrated by the BioTAC system, which can identify direct and complex small molecule binding proteins (78). Coupled with genetic code expansion techniques, PL-MS has facilitated the study of histone post-translational modifications and their interactomes in living cells, offering new insights into epigenetic regulatory pathways (79). The ongoing development of new PL-MS methods, including BioID-based, APEX-based, and photo-activatable techniques (Table 2), combined with their successful deployment across various model systems and research areas, is expected to continue to advance our understanding of cellular dynamics and molecular mechanisms.

### XL-MS

**Figure 3:**
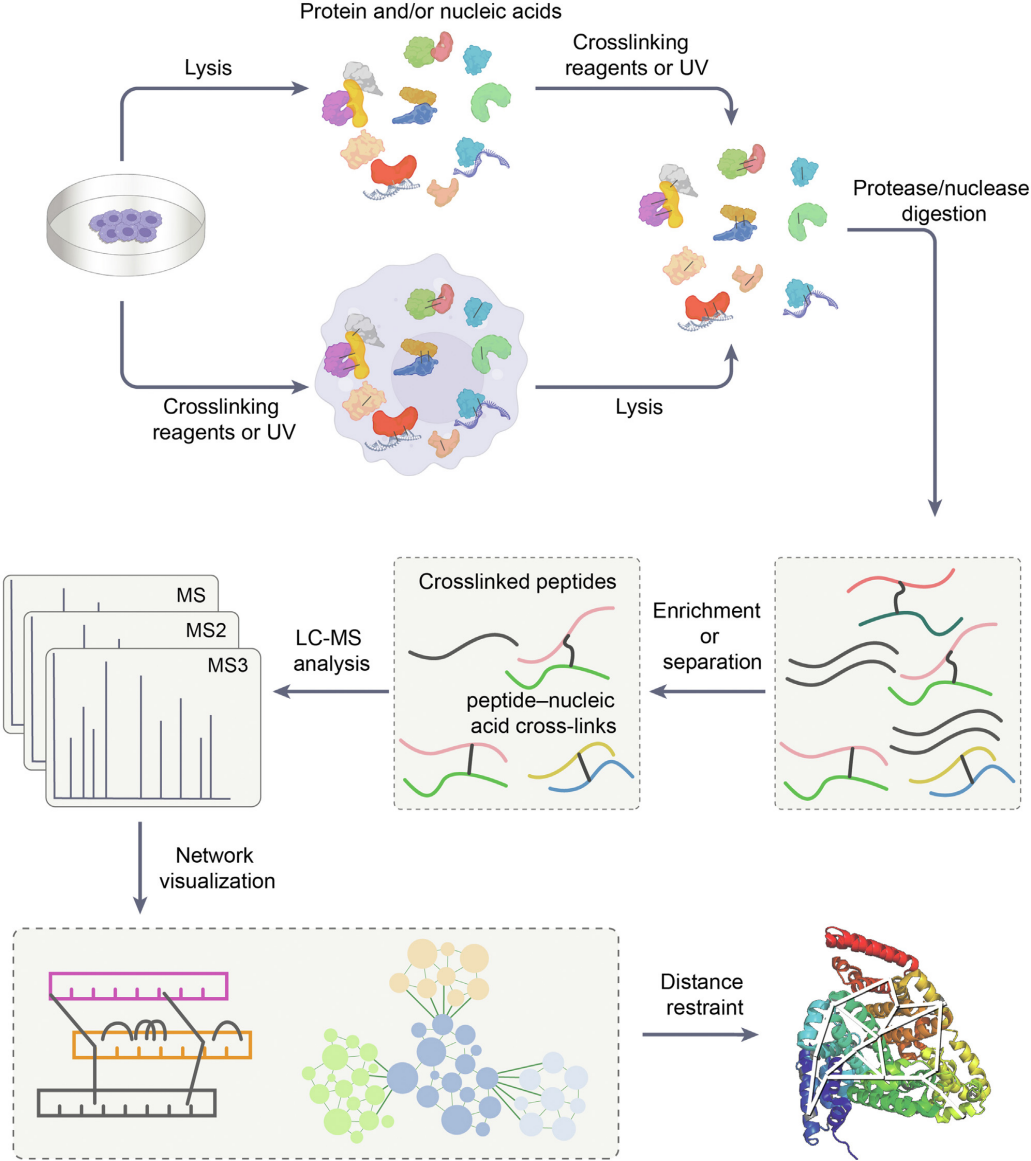
**The workflow of XL-MS.** The workflow begins with a sample containing proteins and/or nucleic acids, which is treated by appropriate cross-linking reagents or ultraviolet (UV) light, depending on the desired class of cross-linking. The choice of cross-linking reagent is based on its functional group's ability to form covalent bonds with components in the sample. These reagents are introduced during the incubation period to mediate the formation of covalent cross-links between interacting proteins or between proteins and nucleic acids, thereby stabilizing these complexes. Alternatively, UV irradiation can also lead to covalent cross-linking between proteins and nucleic acids. The workflow allows for two common approaches: either cross-linking followed by cell lysis, or cell lysis followed by cross-linking of the lysate. After cross-linking, the sample undergoes enzymatic digestion by proteases and/or nucleases to create cross-linked peptide complexes and protein-nucleic acid cross-links, which represent stable interactions between the components of interest. These cross-linked peptides or peptide-nucleic acid complexes may then be enriched or separated from non-cross-linked peptides. The resulting cross-linked samples are analyzed by LC-MS/MS to identify interacting partners and potentially obtain structural information about the complexes. Subsequent data analysis involves MS, MS2 and MS3 scans for the identification of cross-linkers, followed by network visualization to interpret the protein interaction landscape. Additionally, the cross-linking data can be used as distance restraints in molecular modeling to generate structural models of the proteins, protein complexes or protein-nucleic acid interactions.

In recent years, cross-linking-mass spectrometry (XL-MS) has emerged as a technique that bridges structural biology and systems biology by elucidating protein interactions as well as protein conformations and structural dynamics within native cellular contexts. Unlike AP or PL approaches that require a specific bait, there is no such pre-requisite for XL-MS and CF-MS, thus enabling a global exploration of protein networks and structural landscapes without the need for genetic manipulation. A typical XL-MS procedure involves the covalent bonding of proximal amino acid residues and nucleotides within or across proteins and nucleic acids using bifunctional cross-linking agents or ultraviolet (UV) light, followed by enzymatic digests of covalently connected proteins, enrichment of cross-linked peptides, LC-MS/MS analysis, and computational identification of cross-linked peptides (32, 80). In the general procedure, XL-MS can be applied at various stages of sample preparation, each offering unique advantages. *In vivo* cross-linking utilizes membrane-permeable cross-linkers or photo-activatable amino acids to capture interactions in their native cellular environment, preserving transient and weak interactions. *In vitro* cross-linking can be performed on crude cell lysates, allowing for a global view of protein interactions immediately after cell lysis; after fractionation, enabling focused analysis of specific cellular compartments or complexes; or on purified complexes, providing detailed structural insights into specific protein assemblies. This versatility allows researchers to investigate protein interactions at different levels of cellular organization and complexity. The resulting mass spectra can be used to identify the sequences of the crosslinked peptides and map the cross-link positions by specific software tools for automated data processing of cross-links (Fig. 3). These XL-formed linkages can be used as distance restraints to facilitate protein 3D structural modeling, protein subunit topological mapping, and characterization of unknown protein-protein interactions. This method has been increasingly recognized for its capacity to capture transient and elusive interactions that elude traditional purification methods.

The breathtaking revolutionary developments of many novel crosslinking reagents with various chemical specificities that have triggered the simultaneous developments of intelligent MS acquisition methods and software tools by many groups have dramatically advanced the study of global PPIs and the success of other XL-MS applications. The choice of cross-linker is crucial in XL-MS experiments. NHS ester-based cross-linkers, such as disuccinimidyl suberate (DSS), and bis(sulfosuccinimidyl)suberate (BS3), are the commonly used class, primarily targeting lysine residues (and to a lesser extent, threonine, tyrosine, and serine) (32). Formaldehyde (FA), a small and highly permeable molecule, forms cross-links in structured proteins through a 24 Da reaction, with a characteristic MS/MS fragmentation pattern allowing for specific identification and retrieval of structural information in native cellular environments (81). Zero-length cross-linkers, such as EDC, directly conjugate carboxyl groups to primary amines without introducing a spacer (82). MS-cleavable cross-linkers with membrane permeability such as disuccinimidyl sulfoxide (DSSO) and disuccinimidyl dibutyric urea (DSBU) are excellent examples of the developments of the intelligent MS2-MS3 acquisition method and associated software tools such as XlinkX, MeroX and MaXLinker (35, 83, 84). The MS2-cleavable XL-MS method has been recently combined with TMT allowing for multiplex quantitative PPI study of complex biological systems (85). These features are particularly valuable and pivotal as the field is rapidly moving to proteome-wide XL-MS studies.

However, a big drawback of the previous crosslinkers is the detection bias to high abundance proteins over low abundance proteins that are often lost in the presence of a high abundance of peptides in a global scale XL-MS (86). To overcome this limitation, some trifunctional cross-linkers like PhoX have been introduced by adding a third group that allows the selective enrichment of cross-linked peptides prior to MS analysis (87, 88). IMAC affinity-enrichable PhoX is the non-cleavable linker that has been extensively reviewed previously (32, 89). Recently, with the latest development of the isobaric quantitative protein interaction reporter (iqPIR) technology that employs stable isotopes incorporated into the cross-linker, which delivers great benefits of multiplex, accurate quantitation and enhanced detection sensitivity as iTRAQ/TMT do in traditional quantitative proteomics for cross-linked peptides of complex samples, providing deeper insights into protein abundance and modification levels (90). Furthermore, the development of another novel trifunctional bis(succinimidyl) with a propargyl tag (BSP), has improved the efficiency and versatility of XL-MS (91). BSP features a compact size and suitable amphipathic and enrichment capacity, enabling efficient *in vivo* cross-linking within minutes and enhanced cell membrane permeability. Moreover, click-chemistry-based enrichable cross-linkers with cleavable properties (cliXlink), compatible with enrichment strategies have also been developed to facilitate the detection of low-abundance cross-linked peptides, enhancing the sensitivity of interactome analyses, allowing for accurate crosslink identification and providing insights into cellular states in health and disease (92).

Many customized software tools have been developed in different labs as part of specific XL-MS analysis workflow used for identifying cross-linked peptides from fragmentation spectra. The main challenge for the algorithms integrated within each software tool is to project accurate error estimation for reliably estimating false discovery rates (FDRs) and determining the threshold of the results identified by XL-MS. This challenge arises from the unique complexity of XL-MS data, where the combinatorial nature of cross-linked peptide pairs significantly increases the search space compared to traditional proteomics (29). As a result, a typical target decoy approach may not fully fit with cross-linking spectrum matches, and therefore, an additional FDR control at the PPI level is recommended (29, 93). A recent study developed a carefully controlled large-scale analysis using *E. coli* cell lysate, demonstrating that FDRs for PPIs can be reliably estimated and presenting an interaction network comprising 590 PPIs at 1% decoy-based PPI-FDR (29). Our MaxLinker software implemented with a novel “MS3-centric” approach for cross-link identification at 1% FDR allows for significantly reducing the high rate of misidentified cross-links suffered by traditional software tools with “MS2-centric” approach (84). These recent advancements in XL-MS have addressed several previous limitations by introducing trifunctional cross-linkers, isobaric quantitative techniques, and computational approaches (Table 3).

**Table 3:** XL-MS techniques for protein interactomics

Variations	Features	Strengths	Limitations	Use cases	Ref
MS-cleavable XL-MS	Uses MS-cleavable crosslinkers	MS-cleavable crosslinkers offer simplified data analysis	Requires specific fragmentation methods for MS-cleavable	Large-scale interactome studies	(35, 83, 84)
Quantitative XL-MS	Includes TMT-based XL-MS, iqPIR	Enables multiplexed quantitative PPI studies	More complex sample preparation and analysis	Comparative and quantitative interactomics	(85, 90)
Enrichable XL-MS	Includes PhoX, BSP, cliXlink	Selective enrichment of crosslinked peptides; enhanced sensitivity for low-abundance crosslinks	More complex crosslinker chemistry and protocols	Studying low abundance interactions; rapid *in vivo* interactome mapping	(87, 88, 91, 92)

As an example of system-wide XL-MS applications, a notable recent application of XL-MS has been applied for expanding the yeast interactome, identifying a total of 2052 unique residue pair cross-links by DSSO crosslinking with MS2-MS3 and XlinkX analysis pipeline and revealing almost half of the interactions were previously undetected by conventional two-hybrid or AP-MS methods (94). To streamline XL-MS workflows for proteome-wide analyses, a simple workflow based on cross-linker DSBU has been introduced, which allows system-wide protein-protein interaction mapping within a week (95). This optimized methodology has been successfully applied to map interaction networks in *Drosophila melanogaster* embryos, revealing significant insights into the proteomic architecture by identifying thousands of unique cross-linking sites and distinguishing interprotein from intraprotein cross-links. We have employed the DSSO-based MS2-MS3 analysis for domain topological mapping of the bacterial histidine kinase CheA and its 3D structural modeling of the ternary complex with additional chemoreceptor Tar foldon and the coupling protein CheW (96). Advances in XL-MS have also facilitated the characterization of proteome-wide interactions in living cells. For example, an advanced *in vivo* XL-MS platform has been developed for studying dynamic multiprotein complexes in various bacterial compartments. This platform facilitated the identification of approximately 3300 cross-links from the LC-MS/MS analysis of a biological triplicate (20). Besides, a robust *in vivo* XL-MS platform has been created, integrating a multifunctional MS-cleavable cross-linker with advanced sample preparation strategies and high-resolution mass spectrometry. This platform identifies 13,904 unique lysine-lysine linkages from cross-linked HEK 293 cells, facilitating the construction of the largest *in vivo* PPI network known to date, comprising 6439 interactions among 2484 proteins (97). Moreover, a quantitative XL-MS approach using a multiplexed isobaric quantitative cross-linking strategy (6-plex iqPIR) was applied to investigate the effects of Hsp90 inhibitors on the interactome of breast cancer cells, revealing large-scale protein conformational and interaction changes specific to the molecular class of the inhibitors (98).

The principle of covalently linking interacting molecules in XL-MS, which has been effective in studying protein-protein interactions, has found fruitful applications in exploring protein-nucleic acid interactions. Techniques such as UV cross-linking and robust ribonucleoprotein (RNP) capture protocols have been utilized to uncover the SARS-CoV-2 RNA interactome, identifying 109 host factors directly binding to SARS-CoV-2 RNAs (99). The HyPR-MS method has been used to capture and analyze the c-Myc mRNA protein interactome *in vivo*, identifying 229 c-Myc mRNA-binding proteins and suggesting new interactors (100). Similarly, a photocatalytic cross-linking (PhotoCAX) strategy was developed and coupled with MS and RNA sequencing to profile protein-RNA interactions in living cells, revealing 2044 RNA-binding proteins (RBPs) in human HEK293 cells (22). These advancements in XL-MS techniques, including MS-cleavable XL-MS, quantitative XL-MS, and enrichable XL-MS (Table 3), have significantly enhanced our ability to study protein-protein and protein-RNA interactions in native cellular environments.

### CF-MS

**Figure 4:**
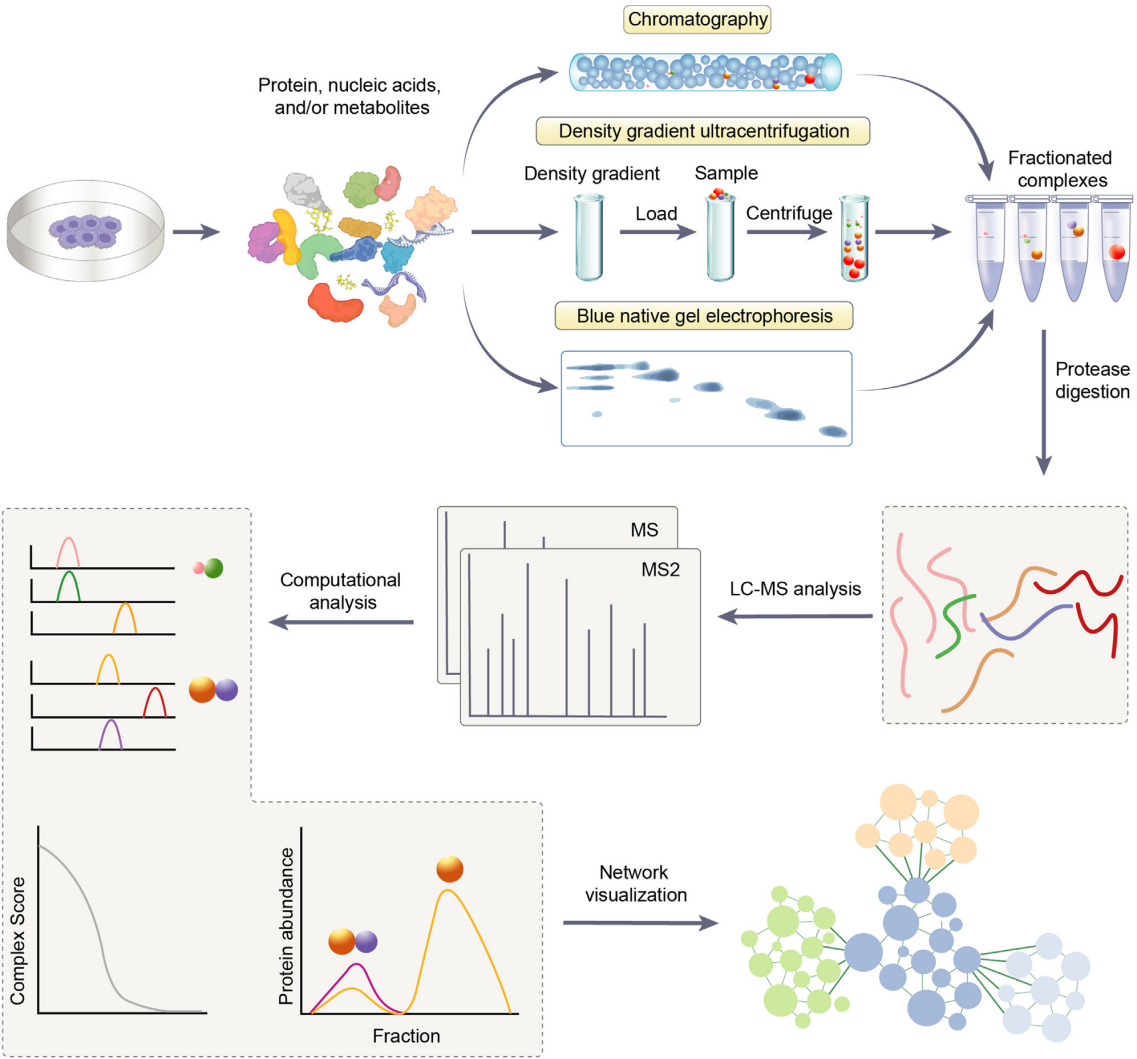
**The workflow of CF-MS.** The process begins with cell lysis to release proteins, nucleic acids, and/or metabolites. This complex mixture is then subjected to fractionation methods to separate the components based on their physicochemical properties. Three fractionation methods are commonly used: native chromatography, density gradient ultracentrifugation, and *blue* native gel electrophoresis. In native chromatography, typically size-exclusion chromatography, the sample is passed through a column where components are separated based on their size and shape. Density gradient ultracentrifugation involves loading the sample onto a density gradient and centrifuging it, resulting in the separation of components into distinct layers based on their density. *Blue* native gel electrophoresis separates complexes in a native gel based on their size and shape, similar to size exclusion chromatography. Following separation by any of these methods, the resulting complexes are fractionated into different fractions, each containing distinct sets of interacting molecules. These fractions then undergo further processing depending on the type of molecules being analyzed. For protein analysis, fractions undergo protease digestion to generate peptides for subsequent LC-MS/MS analysis. The mass spectrometry data is processed to identify proteins, and computational analysis is performed to determine co-fractionation patterns. This analysis typically involves creating elution profiles for each molecule across the fractions, calculating correlation scores between pairs of molecules, and generating abundance distributions across fractions (106). The results are then visualized through network representations, allowing for the identification and characterization of protein interactions based on their co-fractionation behavior.

In addition to XL-MS, co-fractionation-mass spectrometry (CF-MS) has emerged as another powerful technique for the comprehensive mapping of endogenous macromolecular networks on a proteome-wide scale. CF-MS leverages the principle that interacting molecules tend to co-elute during separation techniques under native conditions, thus enabling the inference of protein interactions. In a typical CF-MS workflow, cellular extracts undergo fractionation based on the size or composition of macromolecular assemblies, employing techniques such as native chromatography, density gradient ultracentrifugation, or blue native gel electrophoresis (Fig. 4). Size-exclusion chromatography (SEC) is a commonly used and often the initial method of choice for CF-MS studies (6). In SEC, protein complexes are separated based on their hydrodynamic radius as they pass through a column packed with porous beads. Larger complexes elute earlier, while smaller proteins and complexes are retained longer in the column pores. This technique is particularly effective for preserving native protein interactions and separating complexes across a wide molecular weight range. Other fractionation techniques can also be employed, either alone or in combination with SEC, to enhance separation and provide complementary information. These include ion-exchange chromatography (IEX), which separates complexes based on their net surface charge; density gradient ultracentrifugation, which separates based on the density of complexes; and blue native gel electrophoresis, which maintains native protein interactions while separating complexes primarily by size (6, 101, 102). The choice of fractionation method depends on the specific properties of the complexes under study and the research questions being addressed. The resulting fractions are then analyzed by mass spectrometry to identify the components present in each fraction (103). MS analysis identifies and quantifies the proteins in each fraction, generating elution profiles for proteins and their interacting partners across the fractionation scheme. The co-elution patterns of proteins are subsequently analyzed using sophisticated computational algorithms, which reconstruct protein interaction networks with high confidence (6).

While traditional CF-MS methods have been instrumental in unveiling interactome landscapes, they often suffer from relatively low resolving power for effective isolation of individual macromolecule complexes, experimentally laborious, resource-intensive, and limited quantitative accuracy. To overcome these limitations, a scalable multiplex CF-MS (mCF-MS) platform has been recently developed, enabling the simultaneous measurement and comparison of multi-protein assemblies across different experimental samples at an accelerated rate (18). This approach has been successfully applied to map the protein interaction landscape of non-transformed mammary epithelia versus breast cancer cells, revealing large-scale differences in protein-protein interactions and the relative abundance of associated macromolecules connected with cancer-related pathways and altered cellular processes (18). Further advancements in CF-MS have been achieved by integrating data-independent acquisition (DIA) with CF-MS, resulting in a CF-DIA-MS approach (103). This integration mainly takes advantage of recently enhanced DIA techniques and improves data quality and network generation through extended protein coverage, reduced missing data, and decreased noise. CF-DIA-MS shows promise in expanding our knowledge of interactomes, particularly for non-model organisms.

To enhance the computational analysis of CF-MS data, several advanced methods have been developed. A feature extraction-free approach called SPIFFED (Software for Prediction of Interactome with Feature-extraction Free Elution Data) employs an end-to-end learning architecture that integrates feature representation from raw CF-MS data and interactome prediction using convolutional neural networks, outperforming traditional methods in predicting protein-protein interactions (104). Another approach, EPIC (Elution Profile-based Inference of Complexes), combines supervised machine learning with network analysis to predict protein complexes. EPIC integrates experimental co-elution profiles with functional evidence from public databases to create probabilistic protein-protein interaction networks, which are then clustered to define high-confidence complexes. A key advantage of EPIC is its ability to process both isotope-labeled and label-free CF-MS data, increasing its versatility across different experimental setups (105). PCprophet takes a different approach by utilizing a Random Forest classifier to predict protein complexes directly from CF-MS data, eliminating the need for prior protein-protein interaction inference and allowing for the discovery of novel protein complexes. Furthermore, PCprophet introduces a Bayesian method for differential analysis of complex abundance and assembly state across various experimental conditions, providing insights into dynamic changes in the interactome (106). These advanced computational methods represent significant improvements in the analysis of CF-MS data, each offering unique advantages in predicting protein-protein interactions and complexes and collectively enhancing our understanding of protein interactomes (Table 4).

**Table 4:** CF-MS methods for global protein interactome analysis

Variations	Features	Strengths	Limitations	Use cases	Ref
Chromatography-based	Includes SEC-MS, IEX-MS	Well-established, good for separating complexes based on size or charge	Limited resolution for similar-sized complexes (SEC); may disrupt some interactions (IEX)	Global interactome mapping; Complementary approaches for comprehensive mapping	(18, 107)
Other separation methods	Includes density gradient centrifugation, blue native PAGE	Can handle large complexes, good for membrane proteins; maintains native state	Lower resolution than chromatography methods; limited to smaller complexes for blue native PAGE	Studying membrane protein complexes; Analyzing specific native protein complexes	(40, 101)
Advanced quantitative CF-MS	Includes PCP-SILAM, mCF-MS, CF-DIA-MS	High accuracy in defining complex composition; enables simultaneous measurement across samples; improved data quality	Computationally intensive; requires careful experimental design and specialized setups	Defining precise complex composition; Comparing interactomes across conditions	(18, 103, 108)

Recognizing the potential of CF-MS for comprehensive interactome mapping, large-scale meta-analyses have been conducted to establish better practices for designing and analyzing CF-MS experiments. One study reanalyzed over 200 CF-MS experiments, generating a uniformly processed resource containing millions of protein abundance measurements (27). This resource was used to benchmark experimental designs and optimize computational approaches for network inference, ultimately reconstructing a draft-quality human interactome and predicting over 700,000 protein-protein interactions across multiple eukaryotic species. Furthermore, CFdb, a harmonized resource of interaction proteomics data from 411 CF-MS datasets spanning 21,703 fractions, has been constructed (28). This resource enables meta-analysis of protein abundance, phosphorylation, and interactions across the tree of life, and it includes a reference map of the human interactome. CF-MS has been applied to map protein-protein interactions to encompass diverse research areas. For example, the interactome dynamics of the NF-kappaB pathway in breast cancer reveal pro-tumorigenic interactions and their rearrangement upon NF-kappaB inhibition (107). Additionally, CF-MS has been combined with stable isotope labeling in mammals (PCP-SILAM) to map the interactomes of 7 mouse tissues, providing a proteome-scale survey of interactome rewiring across tissues and identifying systematic suppression of cross-talk between ancient housekeeping and tissue-specific interaction modules (108).

The power of CF-MS to separate and analyze native protein complexes has not only revolutionized our understanding of protein-protein interactions but has also paved the way for exploring other critical molecular interactions. By applying the principles of co-fractionation to diverse biomolecules, researchers have expanded CF-MS to investigate protein-metabolite interactions, offering insights into key regulatory processes in cellular metabolism. PROMIS has provided a comprehensive dataset of protein-small molecule interactions in the model yeast *S. cerevisiae*, uncovering the regulatory role of the Ser-Leu dipeptide in modulating the activity of the glycolytic enzyme phosphoglycerate kinase (8), as well as the role of Tyr-Asp in improving plant tolerance to oxidative stress by directly interfering with glucose metabolism (109). Another approach is MIDAS (mass spectrometry integrated with equilibrium dialysis for the discovery of allostery systematically), which does not employ traditional co-fractionation but utilizes equilibrium dialysis analysis coupled with MS to identify protein-metabolite interactions, has revealed previously unreported interactions between enzymes involved in human carbohydrate metabolism and their metabolite regulators (9). Moreover, CF-MS techniques have been extended to study protein-nucleic acid interactions. The R-DeeP approach utilizes density gradient ultracentrifugation combined with RNase treatment and mass spectrometry to identify and quantify RNA-dependent proteins, providing information on the fraction of a protein associated with RNA-protein complexes (101). This approach facilitates the identification of RNA-dependent proteins, enabling the systematic analysis of RNA-protein interactions on a proteome-wide scale. The adaptability of CF-MS to study various types of biomolecular interactions is reflected in the range of techniques developed within this approach. The variations of CF-MS approaches offer unique advantages for specific experimental needs, expanding the capabilities of CF-MS methods for global interactome analysis (Table 4).

The diverse MS-based approaches discussed above offer complementary insights into the complex landscape of protein interactions, each with its unique strengths in data interpretation. AP-MS and CF-MS excel in providing information on protein complexes and their composition, though they may not always distinguish between direct and indirect interactions. AP-MS typically identifies proteins that are part of the same complex or in close functional association with the bait protein, while CF-MS reveals co-eluting proteins likely to be part of the same complex or share similar physical properties. In contrast, XL-MS offers more direct evidence of binary interactions and spatial relationships between proteins, with cross-linked peptides indicating proximity between specific amino acid residues, either within a protein or between different proteins. This method can provide valuable structural insights through distance restraints. PL-MS occupies a unique niche, offering a snapshot of proteins in close spatial proximity, capable of capturing both stable and transient interactions, though it doesn't necessarily indicate direct physical contact. Proteins identified by PL-MS are within a certain radius of the bait protein, suggesting potential functional relationships or shared cellular localization. When interpreting data from these approaches, researchers should consider the inherent biases and limitations of each method. For instance, while AP-MS and CF-MS are particularly useful for studying protein complexes, they may not provide detailed information about direct physical interactions. XL-MS, while offering more direct evidence of binary interactions, may miss interactions if suitable cross-linking sites are absent. PL-MS data requires careful interpretation to distinguish between functional interactions and mere spatial proximity. Given these varied strengths and limitations, integrating data from multiple complementary approaches often provides the most comprehensive understanding of protein interactions, allowing researchers to build a more complete picture of the intricate cellular interactome.

## Integration of MS-Based Approaches

Previous studies have shown that MS-based methods emerged as powerful techniques for mapping protein interactions and characterizing interactomes. However, each method has inherent strengths and limitations. For example, AP-MS excels in identifying stable and direct protein interactions but often fails to capture weak or transient interactions (41). Conversely, PL methods like BioID and APEX capture stable and transient interactions within defined proximity but cannot distinguish between direct and indirect associations (61, 64). XL-MS can map direct protein-protein contacts with high confidence but struggles to detect weak or dynamic interactions (90). CF-MS provides a comprehensive overview of macromolecular complexes in their native state but lacks the specificity to pinpoint direct interactions (18). Moreover, AP-MS and XL-MS often suffer from low detection sensitivity compared to PL-MS, leading to substantially higher false negatives (61, 110, 111). The main advantage of PL-MS, on the other hand, is its ability to employ biotin affinity enrichment, thereby boosting sensitivity, particularly for mapping protein-protein interactions involving receptor proteins or transcription factors. However, PL-MS can sometimes suffer from a high false positive rate in cases where there are high background levels of endogenous biotinylated proteins (33, 61). Hence, researchers have integrated two or more MS-based methods to overcome these limitations and leverage the complementary strengths of these techniques.

One earlier strategy involves integrating AP and PL-MS, enabling the identification of stable and transient interactions within a single workflow (112). For instance, the MAC-tag approach combines AP-MS and BioID analyses, comprehensively mapping a protein's molecular context (113). The integration of AP and PL-MS has also been successfully applied to study diverse proteins, including transcription factors and the DNA repair scaffold SLX4 (114, 115). Another approach involves combining PL and XL-MS, harnessing the ability of PL to enrich for a specific subcellular compartment and XL-MS to identify direct protein-protein contacts within that compartment (116). This PL/XL-MS strategy has effectively dissected the human nuclear envelope interactome, revealing novel interactions and insights into nuclear matrix organization. Integrating AP and XL-MS has also proven valuable, particularly for studying RNA-protein interactions. The RICK method combines AP with XL-MS and click chemistry to capture and identify RNA-binding proteins interacting with newly transcribed RNAs (10). Similarly, HyPR-MS combines hybridization-based purification of RNA-protein complexes with XL-MS, enabling the characterization of RNA interactomes in tissue samples (117).

Furthermore, researchers have integrated PL and XL-MS to map subcellular interactomes. The APEX-CXMS platform combines APEX proximity labeling with chemical cross-linking and MS, enabling spatially resolved profiling of subcellular protein-protein interactions (118). This approach successfully identified novel interactions between mitochondria and the nucleus. Besides, integration of PL and XL-MS has also been developed to enrich sub-compartment-specific RBPs by integrating peroxidase-catalyzed proximity labeling with organic-aqueous phase separation of crosslinked protein-RNA complexes, which is termed "APEX-PS" (119). Employing APEX-PS, datasets of nuclear, nucleolar, and outer mitochondrial membrane (OMM) RBPs were generated, facilitating the comprehensive identification of RBPs localized to these subcellular compartments. Integration of different MS techniques has also been proven effective for global mapping of the disease-relevant interactomes, such as the Tau interactome in Alzheimer's (120). A combination of APEX proximity labeling, quantitative AP-MS, and proximity ligation assays in the study identified interactions of Tau with presynaptic vesicle and mitochondrial proteins, providing a mechanism for Tau-mediated neurodegeneration. In addition, the combination of AP, PL, and XL-MS has been utilized to comprehensively map interactomes, as demonstrated in the study of the SnRK1 kinase signaling network in plants (121). This multi-dimensional approach revealed fundamental regulatory interactions and provided structural insights into the SnRK1 heterotrimer complex.

In summary, integrating MS-based approaches has significantly expanded our ability to comprehensively map interactomes and elucidate the intricate networks of biomolecular interactions governing cellular functions. These strategies allow the synergizing of the strengths of individual techniques, enabling the capture of diverse interaction types, from stable complexes to transient associations, and providing multi-dimensional insights into biological networks and mechanisms.

## Computation-Based Protein Interactomics

Complementary to experimental techniques, computational approaches allow high-throughput and inexpensive ways to investigate the complicated networks of biomolecular interactions. Computational tools have revolutionized the study of protein interactomics, from predicting protein interactions and modeling protein and complex structures in computational biology to integrating computational methods with MS-based techniques. By combining these disparate data types, such as sequence and structural information, interactome data, and functional networks, computational tools have revealed novel biological insights and opened new avenues for applications.

### Protein Interaction Predictions

Mapping the vast networks of protein interactions governing cellular processes places a premium on protein interaction prediction methods. As the mysteries of biological systems unfold, the advanced predictive tools for unraveling them become increasingly paramount. These methods encompass a wide array of approaches, ranging from computational and machine learning techniques to experimental and hybrid strategies, all being designed to identify interaction partners, characterize the dynamics of these interactions, and predict how alterations in these interactions could influence biological outcomes. Network-based methods for PPI prediction have emerged as particularly valuable tools in interactomics, offering researchers the ability to predict potential interactions on a large scale, complementing experimental techniques, and guiding further investigations. The International Network Medicine Consortium has recently spearheaded significant efforts to systematically evaluate and benchmark network-based methods for PPI prediction across diverse organisms, including *A. thaliana*, *C. elegans*, *S. cerevisiae*, and *Homo sapiens* (122). This study utilized a rigorous methodology involving benchmarking 26 representative network-based methods. Through extensive computational and experimental validations, the results showed that advanced similarity-based methods, which leverage the underlying network characteristics of PPIs, show superior performance over other general link prediction methods in the examined interactomes (122). This benchmarking study provides researchers with a valuable framework for selecting appropriate prediction methods based on their specific research context and organism of study, thereby advancing our ability to unravel the complexities of protein interaction networks.

Recent developments have also introduced more specialized prediction tools. For instance, NECARE, a network-based method incorporating knowledge-based features and relational graph convolutional networks, was developed to predict PPIs specific to cancer (123). This approach has enabled the mapping of cancer interactome perturbations and the identification of cancer hub genes, providing insights into potential therapeutic targets and prognostic markers. To improve interaction prediction by incorporating protein structure information, AlphaPulldown was developed (124). This method enhances PPI prediction by utilizing high-quality structural models of protein complexes generated with AlphaFold-Multimer. AlphaPulldown offers a command-line interface, confidence scores, and tools for graphical analysis. It also simplifies the discovery of new PPIs by providing streamlined access to the structural modeling of protein complexes with unprecedented accuracy. In addition to structure-based and network-based approaches, quantitative feature-based methods have been explored for PPI prediction. A framework employing Boruta feature selection, Monte Carlo feature selection, and incremental feature selection was developed to determine significant protein attributes like stoichiometric balance, protein abundance, molecular weight, and charge distribution (125). It incorporates a quantitative decision-rule system using algorithms like random forest and RIPPER to assess potential interactions under realistic conditions, offering new insights into the complex biological mechanisms governing PPIs.

For PNI prediction, methods like dMaSIF, which employ geometric deep learning frameworks, have effectively identified nucleic acid binding sites and predicted interactions. These approaches can facilitate the investigation of RNA-binding proteins and their roles in various biological processes (126). CLAPE, a novel approach integrating pre-trained protein language models and contrastive learning, has demonstrated superior performance and generalization ability in predicting DNA binding residues (127). This framework thus holds great potential for applications in studying the intricate interactions between proteins and DNA. Integrating molecular structure and interactome data has additionally been explored using deep learning frameworks to predict PMI (128). These methods have improved prediction accuracy and robustness, highlighting the synergistic effects of combining different data sources and capturing perspectives beyond sequence homology and chemical similarity. The notable development is the emergence of machine learning and deep learning approaches for predicting protein-ligand binding. AI-Bind, a pipeline that combines network-based sampling strategies and unsupervised pre-training, has demonstrated improved binding predictions for novel proteins and ligands (129). This high-throughput approach holds great promise for accelerating drug discovery by identifying potential drug-target combinations and active binding sites within amino acid sequences.

The array of protein interaction prediction methods, including network-based approaches, machine learning techniques, and structure-based predictions, provide researchers with powerful tools to explore protein interactions. These computational methods are valuable for hypothesis generation, interactome completion in understudied systems, cross-species predictions, and prioritizing experimental targets. Specialized tools like NECARE enable context-specific predictions, while AlphaPulldown integrates structural information to assess interaction probabilities. However, these prediction methods should complement, not replace, experimental techniques. Their true potential is realized when used in conjunction with experimental validation and other computational strategies, forming a comprehensive approach to mapping protein interaction networks. Researchers should employ these predictive tools as part of an integrated strategy, using them to guide experimental design, refine hypotheses, and supplement empirical findings in their efforts to unravel cellular interactomes.

### Protein Structure and Complex Modeling

While mass spectrometry-based techniques provide crucial experimental data on protein interactions, computational approaches for protein structure and complex modeling offer complementary insights into the mechanisms underlying these interactions and play a vital role in elucidating the structural basis of protein-protein interactions and complexes identified through MS-based interactomics studies. Recent years have seen revolutionary advances in computational approaches for protein structure prediction, particularly with the development of deep learning-based methods. These advancements have dramatically improved our ability to predict three-dimensional structures of proteins and their complexes, providing crucial insights into molecular mechanisms of protein interactions.

A notable example of this progress is AlphaFold, a neural network-based model that has achieved near-atomic accuracy in protein structure prediction (130). AlphaFold's architecture integrates multiple components that contribute to its high accuracy, including a sequence module that processes multiple sequence alignments (MSAs) to infer evolutionary relationships and potential residue contacts, and a structure module that refines structural predictions using physical and geometric constraints. While MSAs provide valuable evolutionary information, it's important to note that AlphaFold can predict correct structures even with limited MSA data. This remarkable achievement has opened new avenues for large-scale structural bioinformatics and mechanistic understanding of protein function. CombFold, a combinatorial and hierarchical assembly algorithm, has been developed for large protein assemblies to predict structures by utilizing pairwise interactions between subunits predicted by AlphaFold2 (131). This method has proven highly effective in accurately predicting large and asymmetric protein assemblies, thus broadening the structural insights into multimeric proteins. The accuracy of structural predictions has been further refined by improvements in multiple sequence alignment (MSA) construction. DeepMSA2, an iterative alignment pipeline, sifts through genomic and metagenomic sequence databases to produce refined MSAs for protein monomers and complexes (132). These enhanced MSAs have significantly improved the precision of both tertiary and quaternary structural predictions, emphasizing the critical role of input quality in deep learning-driven models. Complementing these deep learning-based methods, language models have also shown remarkable capabilities in predicting protein structures directly from primary sequences. The ESMFold model, a large language model trained on protein sequences, can infer full atomic-level protein structures from sequence data alone (133). This approach has enabled the construction of the ESM Metagenomic Atlas, a comprehensive database of predicted structures for over 617 million metagenomic protein sequences, providing a rich resource for studying the diversity of natural proteins.

Expanding the scope of predictive models, RoseTTAFoldNA has extended the capabilities of structure prediction to protein-nucleic acid complexes (134). This single-trained network can rapidly produce high-accuracy three-dimensional structure models with confidence estimates for protein-DNA and protein-RNA complexes, providing insights into these critical biological complexes. Meanwhile, DynamicBind has emerged as a deep learning method that employs equivariant geometric diffusion networks to predict ligand-specific protein-ligand complex structures (135). By constructing a smooth energy landscape, DynamicBind can accurately recover ligand-specific conformations from unbound protein structures, facilitating the identification of cryptic pockets and thereby accelerating computational drug discovery.

At a broader level, deep learning methods like AlphaFold three and RoseTTAFold All-Atom (RFAA) have been developed to model a wide range of biomolecular interactions, including those between proteins, nucleic acids, small molecules, metals, ions, and covalent modifications (31, 136). AlphaFold 3, with its substantially updated diffusion-based architecture, demonstrates significantly improved accuracy over many previous specialized tools in predicting protein-ligand, protein-nucleic acid, and antibody-antigen interactions, enabling high-accuracy modeling across biomolecular space within a single unified deep learning framework (136). Similarly, RFAA combines a residue-based representation of amino acids and DNA bases with an atomic representation of all other groups, allowing it to model the structures of diverse biomolecular assemblies given their sequences and chemical structures (31). These groundbreaking computational methods have revolutionized our approach to studying biomolecular systems. For example, we have previously integrated protein structural modeling with other techniques to investigate the interaction mechanisms between intrinsically disordered proteins and protein-ligand complexes, providing significant new insights into the functions of these proteins (39, 137, 138). Furthermore, recent advances in computational methods are opening new avenues for exploring complex biological structures and designing novel proteins with tailored functionalities. These developments are enhancing our understanding of molecular interactions at the systems biology level. By providing atomic-level details of protein structures and complexes, these computational approaches enable researchers to interpret MS-based interactomics data in a structural context. This integration of structural information with interactomics data allows scientists to formulate hypotheses about interaction mechanisms, design targeted experiments for validation, and enable the creation of innovative solutions in biological research.

### Integration of Computational Tools With MS

Integrating computational tools with MS-based techniques has unlocked new frontiers in the comprehensive study of protein and structural interactomes. By synergizing the strengths of these complementary approaches, researchers can overcome the limitations of the individual methods and gain unprecedented insights into the intricate networks of biomolecular interactions. One prominent application lies in the integration of structural information with interactomics data. Proteo3Dnet is a web server that capitalizes on integrating mass spectrometry-based interactomics data with structural information (139). It operates by employing several computational strategies: (i) identifying interologs that have resolved structures in the Protein Data Bank, including cross-species homology searches; (ii) predicting transient interactions mediated by Short Linear Motifs (SLiMs) using the ELM database (140) and (iii) referencing physically validated interactions from the BioGRID database (141). By amalgamating these diverse data sources, Proteo3Dnet constructs structural interaction networks from interactomics data, offering a detailed and dynamic view of potential protein-protein interactions and suggesting possible undiscovered partners or condition-specific binding events. This integrated approach helps researchers visualize the complex web of interactions at a structural level, enhancing the understanding of biological processes and interactions. Building upon the integration of structural and interactomics data, recent studies have further expanded the synergy between computational tools and MS-based techniques. For instance, the combination of CF-MS, AlphaFold, and cryo-EM has been demonstrated as an effective approach for identifying protein complexes in native cell extracts from *S. cerevisiae*. This approach successfully identified and structurally characterized the 2.6 MDa complex of yeast fatty acid synthase, highlighting the potential of multi-technique integration in elucidating complex cellular structures (142).

Additionally, computational tools have been developed to analyze and visualize MS-based interactomics data. For example, CANVS is an easy-to-use application that cleans, analyzes, and visualizes interactome/association data, integrating systems biology databases and providing Gene Ontology tools for contextualizing results (143). Notably, Tapioca, an ensemble machine learning framework, is designed to predict novel protein interactions by integrating mass spectrometry interactome data, protein properties, and tissue-specific functional networks (21). Tapioca leverages diverse mass spectrometry data sources, including thermal proximity coaggregation (TPCA), ion denaturation, and co-fractionation workflows. In particular, TPCA works on the principle that interacting proteins stabilize each other when exposed to thermal denaturation. In this workflow, intact cells are subjected to a temperature gradient, followed by lysis of each fraction, with soluble portions labeled using tandem mass tags (TMT) for multiplexed MS analysis. By integrating these diverse data, Tapioca has been applied to investigate viral infection dynamics, revealing proviral hub proteins and broader interactome rewiring.

Furthermore, integrative modeling techniques have been developed to combine XL-MS data with computational methods for protein structure prediction. For instance, HADDOCK, a leading integrative docking and modeling platform, incorporates XL-MS restraints into protein-protein and protein-ligand docking simulations. This advancement has significantly enhanced the accuracy of predicted protein complex structures, allowing both experts and non-experts to explore intricate macromolecular assemblies (144). Additionally, targeted *in situ* XL-MS was employed to probe the structures of SARS-CoV-2 proteins Nsp1, Nsp2, and nucleocapsid (N), followed by integrative modeling that computationally combined the cross-linking data with domain structures predicted by AlphaFold2 (145). This approach generated full-length atomic models, revealing insights into these proteins' topology and potential physiological functions. Building on this, AlphaLink incorporates experimental distance restraint information, such as photo-crosslinking data obtained through in-cell MS, into the AlphaFold2 architecture, enhancing the prediction accuracy for challenging targets that undergo conformational changes or lack homologous sequences (146). This noise-tolerant framework exemplifies the synergy between experimental data and advanced deep-learning models in accurately characterizing protein structures from in-cell data.

Moreover, the integration of XL-MS data with computational tools has also proven valuable in understanding protein dynamics and structural heterogeneity. Short-distance cross-linking data were utilized as restraints in discrete molecular dynamics simulations, successfully predicting the structures of myoglobin and FK506 binding protein (147). Additionally, a study on the *E. coli* ribosome using the cross-linker disuccinimidyl diacetic urea (DSAU) revealed insights into flexible regions, conformational changes, and stoichiometric variations in bound ribosomal proteins, demonstrating the power of XL-MS in guiding structural refinement and identifying regions of flexibility in complex biomolecular assemblies (148). A pipeline combining AlphaFold2 ensemble generation and conformer selection using monolink and crosslink probability scores based on residue depth has successfully identified open and closed conformations of proteins like Complement component 3, luciferase, and glutamine-binding periplasmic protein (149). This highlights the complementarity of AlphaFold2 with experimental XL-MS data in resolving structural heterogeneity. The largest XL-MS dataset to date was presented in a landmark study, capturing 28,910 unique residue pairs across 4084 human proteins and 2110 protein-protein interactions (17). By integrating XL-MS data with AlphaFold2 predictions, they demonstrated the power of this approach in experimentally assessing protein and complex structures and evaluating and corroborating in silico predictions, enabling comprehensive mining of the structural proteome and interactome. These studies revealed that integrating computational tools with MS-based techniques has catalyzed groundbreaking advancements in studying protein and structural interactomes.

## Future Perspectives

The rapid advances in MS-based techniques, including AP-MS, PL-MS, XL-MS, and CF-MS, and computational tools have revolutionized the field of protein interactomics, enabling unprecedented insights into the intricate networks of biomolecular interactions that govern cellular processes. The integration of these cutting-edge approaches has been proven to be a powerful synergy, overcoming the limitations of individual methods and providing an extended understanding of interactome landscapes. Building on these foundational advances, researchers continue to refine and expand the capabilities of MS-based techniques, pushing the boundaries of interactome exploration even further.

Looking towards the future, it can be anticipated that several exciting avenues will likely emerge. The continued development of cross-linking reagents and enrichment strategies will further enhance the sensitivity, quantitation, and coverage of XL-MS, enabling the capture of low-abundance and transient interactions in an accurate quantitative manner. Integrating multi-omics data, such as transcriptomics and metabolomics, with interactomics data holds the potential to unravel the intricate interplay between various biomolecular networks (7). The advent of single-molecule and single-cell interactomics techniques will provide unprecedented resolution, enabling the dissection of heterogeneous cell populations and the investigation of rare cellular events. Integrating computational tools with MS-based techniques has catalyzed groundbreaking advancements in studying protein interactomes and structural interactomes, but the current level of integration remains limited. Hence, the synergistic integration of computational tools and MS-based techniques is expected to accelerate. For example, although initiatives like Proteo3Dnet and AlphaLink represent pioneering efforts, the integration of these advanced methodologies remains at an early stage (139, 146). There is a pressing need for deeper and more comprehensive integration strategies to fully harness the synergistic potential of MS-based techniques and computational tools. Future efforts should develop unified frameworks seamlessly incorporating diverse MS data types, including AP, PL, XL, and CF, into advanced computational pipelines for interaction prediction, structural modeling, and dynamics simulations. The development of specialized machine learning models trained on interactomics data, coupled with advanced structural prediction algorithms, will facilitate the rapid identification of novel interactions and the elucidation of their structural underpinnings (129). Integrating generative models and physics-based simulations could enable the rational design of proteins with tailored functions and the exploration of hypothetical interactomes (31). Nevertheless, such integration could leverage the strengths of both approaches, enabling the construction of highly accurate structural interactome networks, elucidating protein complex dynamics, and investigating condition-specific or disease-related interactome perturbations.

The impact of these advancements extends beyond fundamental research, holding significant implications for various applications, including drug discovery, disease diagnostics, and bioengineering. The comprehensive mapping of disease-specific interactomes and identifying key regulatory nodes would pave the way for developing targeted therapeutics and biomarkers (120). The ability to rationally design protein complexes with desired functionalities could revolutionize fields such as biocatalysis, biosensing, and biomaterials engineering. Overall, the field of protein interactomics stands at an exciting juncture, driven by the synergistic advancements in MS-based techniques and computational tools. As these approaches evolve and ultimately merge, we can anticipate profound insights into the intricate networks that orchestrate cellular functions, ultimately leading to transformative discoveries in biology, medicine, and biotechnology.

## Data Availability

All supporting data are provided within the manuscript, supplementary data, and supplementary tables.

## Conflict of Interest

The authors declare that they have no conflicts of interest with the contents of this article.
